# Engineering of Fc Fragments with Optimized Physicochemical Properties Implying Improvement of Clinical Potentials for Fc-Based Therapeutics

**DOI:** 10.3389/fimmu.2017.01860

**Published:** 2018-01-08

**Authors:** Chunpeng Yang, Xinyu Gao, Rui Gong

**Affiliations:** ^1^CAS Key Laboratory of Special Pathogens and Biosafety, Wuhan Institute of Virology, Chinese Academy of Sciences, Wuhan, China; ^2^University of Chinese Academy of Sciences, Beijing, China

**Keywords:** monoclonal antibody, Fc-fusion protein, Fc-based therapeutics, optimization, physicochemical property, stability, aggregation

## Abstract

Therapeutic monoclonal antibodies and Fc-fusion proteins are successfully used in treatment of various diseases mainly including cancer, immune disease, and viral infection, which belong to the Fc-based therapeutics. In recent years, engineered Fc-derived antibody domains have also shown potential for Fc-based therapeutics. To increase the druggability of Fc-based therapeutic candidates, many efforts have been made in optimizing physicochemical properties and functions mediated by Fc fragment. The desired result is that we can simultaneously obtain Fc variants with increased physicochemical properties *in vitro* and capacity of mediating appropriate functions *in vivo*. However, changes of physicochemical properties of Fc may result in alternation of Fc-mediated functions and *vice versa*, which leads to undesired outcomes for further development of Fc-based therapeutics. Therefore, whether modified Fc fragments are suitable for achievement of expected clinical results or not needs to be seriously considered. Now, this question comes to be noticed and should be figured out to make better translation from the results of laboratory into clinical applications. In this review, we summarize different strategies on engineering physicochemical properties of Fc, and preliminarily elucidate the relationships between modified Fc *in vitro* and the subsequent therapeutic influence *in vivo*.

## Introduction

Since the hybridoma technology for the production of monoclonal antibodies (mAbs) was invented more than 40 years ago, mAbs are widely used as diagnostics and therapeutics. The first commercial therapeutic mAb, muromonab-CD3 (trade name Orthoclone OKT3), was approved by the U.S. Food and Drug Administration (FDA) for prevention of kidney transplant rejection in 1986 (1). Because it is a murine antibody, administration of this antibody in human might lead to the production of human anti-mouse antibody (HAMA) responses. To reduce the immunogenicity, the development of therapeutic mAbs undergoes four generations: murine mAbs, chimeric mAbs, humanized mAbs, and fully human mAbs (2). Nine therapeutic mAbs have been approved by the U.S. FDA since the start of 2017^1^ (3) while the total sales of 70 mAbs for clinical treatment will exceed 100 billion U.S. dollars this year. Among those mAbs, Adalimumab (trade name Humira) the first approved fully human mAb derived from phage display, led the list of top-selling pharmaceutical products with global sales of 16 billion U.S. dollars in 2016 (4).

Fc-fusion proteins are composed of an immunoglobulin (Ig) Fc domain that is directly linked to another peptide, protein, or protein domain. For therapeutic propose, the first description of CD4-Fc fusion protein showed the inhibitory activity against the formation of syncytia during HIV-1 infection in 1989, which showed the proof-of-concept of use of therapeutic Fc-fusion proteins for treatment of HIV-1 infection (5). Subsequently, many modified CD4-Fc fusion proteins were constructed including PRO 542, which was still clinically evaluated (6, 7). Etanercept (trade name Enbrel), a recombinant human tumor necrosis factor (TNF) receptor-Fc fusion protein, was the first TNF-α antagonist approved in the USA for the treatment of rheumatoid arthritis in 1998 (8, 9). The total sale of etanercept is about six billion U.S. dollars in 2016 reported by Amgen (Chairman and CEO Letter and Amgen Inc. 2016 Annual Report^2^), which demonstrates huge market for therapeutic Fc-fusion proteins.

Fc-derived antibody domains are emerging candidates as Fc-based therapeutics. Since they are part of Fc fragments, they may have complete or partial Fc-mediated functions. Therefore, they could be used as scaffold for selection of functional binders, or as carrier for generation of novel fusion proteins for potential clinical use (10). Several candidates have been selected based on Fc-derived scaffolds while Fc-derived domain-fusion protein were also constructed, which were promisingly functional in the aspect of potential therapeutic significance. However, the gap between current progress and clinical use is still huge.

Although great success has been achieved, there are still many limitations during the development of Fc-based therapeutics from bench to bedside (1, 11). The poor physicochemical properties are considerable factors that lead to failure of potential candidates in clinical trials. For marketed drugs, such drawbacks could increase their adverse effects. Therefore, a new-developed candidate should be well characterized in its physicochemical properties which may need to be optimized for better therapeutic outcomes, while increase of physicochemical properties could also be one strategy to make current biodrugs better (biobetter). Although a lot of effort has been put into modification of physicochemical properties on Fc fragment, whether modified physiochemical properties can lead to desired efficacy *in vivo* has not be well understood yet. Here, we summarize the methodology in engineering of physiochemical properties of Fc and try to discuss how the improvement of physicochemical properties influences the efficacy for administration *in vivo*.

## Fc-Based Therapeutics

As described above, Fc-based therapeutics are now widely used in therapy and prevention of various diseases. Fc is the crystallizable fragment derived from Ig which has five classes including IgG, IgA, IgD, IgM, and IgE in human (12). An Ig molecule is composed of antigen-binding fragments (Fab) Fc. Fc plays multiple roles in dimerization for formation of Y-shaped structure of Ig and maintenance of the structure, and Fc-mediated effector functions and extension of serum half-life. There are two domains: second constant domain (CH2) and third constant domain (CH3) in monomeric Fc of IgG. CH2 domain has a single N-linked glycan at N297 (all the antibody residues here are numbered according to EU numbering (13) unless specified). Two CH3 domains interact strongly with each other to form homodimer resulting in dimerization of Fc. These elements contribute to the physicochemical characteristics of Fc. Through the binding of Fc in IgG to its receptor Fc-gamma receptors (FcγRs), immune leukocytes presenting FcγRs on their surface membrane are recruited and activated, which subsequently triggers antibody-dependent cell-mediated cytotoxicity (ADCC) and antibody-dependent cell-mediated phagocytosis to kill and clear target cells (e.g., tumor cells) (14, 15). In addition, Fc can bind to the serum complement molecule (C1q) to initiate the assembly of membrane attack complex formed by complement cascade proteins to destroy target cells, which is termed complement-dependent cytotoxicity (CDC) (16, 17). These effector functions are important for the pharmaceutical efficacies of Fc-based therapeutics. Besides mediation of effector functions, Fc can also bind to neonatal Fc receptor (FcRn) in a pH-dependent manner, which leads to the extension of the serum half-life of IgG (18). In addition, binding of Fc to immune-related molecules such as Fc receptors can regulate immune response *in vivo* (19). Taking together, Fc part in an Fc-based therapeutic protein plays important roles in biological and pharmacological properties including (i) increased stability and aggregation resistance; (ii) acquired multivalent binding to the target; (iii) enhanced Fc-mediated effector functions; (iv) extended serum half-life; and (v) modulated immunogenicity.

### Full-Size mAbs

The therapeutic mAbs have been successfully used for the treatment of various diseases including cancer, immune disease, and infection (20–22). They are highly specific and efficient. Currently, most of the U.S. FDA-approved therapeutic mAbs are full-size IgG molecules with a molecular weight of approximately 150 kDa. Within the IgG isotype, there are four subtypes (IgG1–IgG4) with differing properties distinguished by different hinge regions and Fc fragments. Most of the currently marketed therapeutic IgGs are of the IgG1 subtype while no mAb of IgG3 format is approved yet. A typical IgG1 molecule contains two copies of Fab fragments and one Fc fragment. Each Fab fragment contains a heavy chain variable domain (VH), a light chain variable domain (VL), a first heavy chain constant domain (CH1), and a light chain constant domain (CL). The Fc fragment is dimeric which contains two CH2 domains and two CH3 domains. Fab and Fc are connected by flexible hinge region. In total, a full-size mAb has 12 Ig-fold domains, which means the formation of corrected folding is a complicated process in the production of functional IgG1 molecule. Therefore, how to prevent them from unfolding and aggregating is still a big challenge in modern biopharmaceutical industry (23).

### Fc-Fusion Proteins

Lots of endogenous proteins in our body and other foreign proteins may have potential to treat various diseases due to the antagonistic or agonistic effects. However, these proteins may have many limitations (e.g., instability and rapid clearance rate from the circulation), which constrains their further application in clinic. To solve this problem, fusion of them to the antibody Fc fragment is an effective strategy. Fc-fusion proteins are molecules in which the Fc fragments are fused to proteins of interests, such as extracellular domains of receptors, soluble cytokines, ligands, enzymes, engineered domains, or peptides (24–27). Therefore, Fc-fusion proteins inherit some antibody-like properties such as relatively good physicochemical characteristics for easy expression, purification, formulation, storage and transportation, bi- or multivalent, long serum half-life, enhanced function, and adjustable immunogenicity, which increases the possibilities for clinic use. However, the similar problems as mentioned in the above paragraph should also be considered during clinical development of Fc-fusion proteins.

### Engineered Domains from Fc Fragment as Potential Therapeutics

A major problem for full-size mAbs is their poor penetration into tissues (e.g., solid tumors) and weak or absent binding to sterically restricted regions on the surface of some molecules (e.g., on the viral envelope glycoproteins), which are fully accessible only by molecules with small size (28). Reduction of molecular weight of full-size mAbs such as identification of the minimum binding domain is one of attractive directions to overcome the drawbacks. Therefore, lots of derivates from intact Igs appeared during last two decades. These derivates include Fab, single chain variable fragment (scFv), heavy chain variable domain (VH) and light chain variable domain (VL) (29). The functional VH from camelidae, specially termed as nanobody, has been clinically developed by Ablynx. However, due to the lack of Fc part, the serum half-lives of these variants are relatively short, which is one of the major obstacles for *in vivo* administration (29). Other Ig domains including CH2 and CH3 derived from Fc and Fc itself have been proposed as new scaffolds for development of novel Fc-based therapeutics (10, 28, 30, 31). They are also smaller than full-size mAbs. Compared with those variants from Fab-based backbone, these scaffolds from Fc-based backbone might offer additional Fc-mediated advantages such as high stability, potential effector functions, and long half-life due to incorporation of full length or truncated Fc fragment (10, 28). Optimization of the Fc-based scaffolds on their folding and aggregation resistance could confer better potentially therapeutic outcomes.

The full-size mAbs, Fc-fusion proteins, and engineered domains from Fc fragment share Fc fragment or portion of Fc fragment as common region. Therefore, engineering of Fc could be benefit for all of them. Here, we focus on the current progress in increase of physicochemical properties of Fc including stability and aggregation resistance toward better clinic consequences.

## Increase of Physicochemical Properties of Fc Fragment

In general, the stability and aggregation resistance of a protein are two major physicochemical properties we most concern. The stability is the ability of a protein which retains its correctly folded conformation under harsh conditions such as high temperature, chemical denaturant, protease, and others, while the aggregation-resistant property is to reduce the formation of soluble oligomers and insoluble precipitates during expression, concentration, storage, and others.

One important restrictive factor in development of Fc-based therapeutics is that these proteins may tend to unfold and aggregate upon exposure to various stresses (32), including agitation (33, 34), high temperature (35, 36), low pH (37, 38), high protein concentration for subcutaneous therapeutic delivery (39), freeze–thaw cycle (40, 41), and transportation and long-term storage (42, 43), which means loss of function and increase of immunogenic risk. For example, aggregation may lead to not only loss of activity but also immune response, and negatively impact on many production processes including expression, purification, and formulation (44). A stable protein typically could be correctly expressed at high level, easily purified with no requirement of specific conditions such as low temperature and additional protease inhibitor, which makes the manufacture process much easier and cheaper. It remains active during long-term storage and after administration *in vivo*, which achieves less frequent usage at a lower dosage. An aggregation-resistant protein could be concentrated to high concentration without formation of inactive oligomer, which reduces the injection volume and makes patient more comfortable. Although stability and aggregation propensity are in different descriptions, the relationships between them are close (45). In many cases, increased stability can also lead to less aggregation propensity. Hence, during the development of recombinant therapeutic proteins, prevention of unfolding and aggregation is essential for ensuring efficacy and safety. The structure of a protein is maintained by covalent and non-covalent interactions (46). The covalent interactions are typically caused by the formation of disulfide bonds between two cysteine residues. The non-covalent interactions include hydrogen bonds, Van der Waals forces, hydrophobic interactions, and salt bridges (ionic bonds). These interactions guide the correct folding of a protein to form secondary structure such as α-helix and β-sheet, then form advanced tertiary structure and quaternary structure. Therefore, optimizing the covalent and non-covalent interactions is major direction to increase the physiochemical properties of Fc fragment.

### Role of CH2 and CH3 in Maintaining Fc Structure

**Figure 1:**
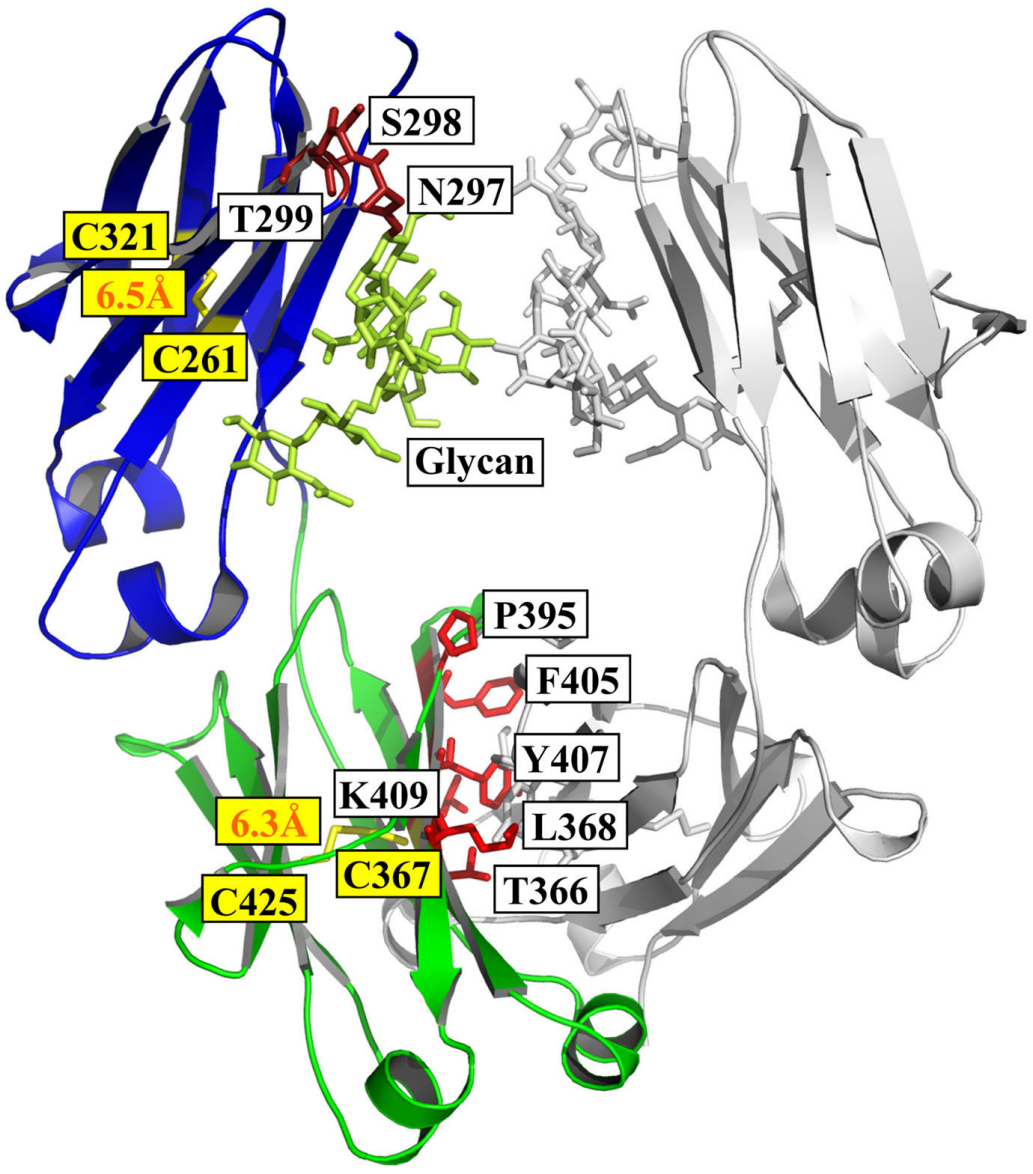
Structure of Fc [PDB 3AVE (47)] presented by PyMOL. The CH2 and CH3 domains are colored by blue and green, respectively; the residues (N297, S298, and T299) in glycosylation motif in CH2 are colored dark red; the residues (T366, L368, P395, F405, Y407, and K409) involved in the interactions between two CH3 domains are colored by light red; and the oligosaccharides and native disulfides are colored by lemon and yellow, respectively.

The antibody heavy chain constant domain is generally defined as CH1–CH2–CH3 in IgG, IgA, and IgD, with an additional domain (CH4) for IgM and IgE (12). The crystal structure of a fucosylated human IgG1 Fc is used here for presenting (PDB 3AVE) (47) (Figure 1).

Structural comparison of fucosylated CH2 in IgG, IgA, and IgD is equal to CH3 in IgM and IgE. The primary sequence of IgG CH2 has a glycosylation motif N297–S298–T299 that results in N-linked glycosylation (48). The oligosaccharides are important for the stability, aggregation propensity, and effector functions of Fc fragment (49). For example, removal of the oligosaccharides results in reduction of aggregation resistance in IgG1 under acidic conditions (50).

The secondary structure of CH2 consists of two β-sheets forming a barrel as solved crystal structure of glycosylated IgG1 CH2 in an intact IgG1 molecule (51), a fucosylated human IgG1 Fc (47), or isolated aglycosylated CH2 (52) (Figure 1). There are seven β-strands from A to G connected by three loops (loops BC, DE, and FG) and two helices (helix 1 and 2) in CH2 (Figure 1). The native disulfide bond between strand B (C261) and F (C321), buried in the hydrophobic core of the molecule, should be important for the structural stability of CH2 although the direct evidence is still lacking (Figure 1). CH2 is relatively unstable compared with other Ig domains such as CH3 (53, 54). For example, during thermo-induced unfolding, the melting temperature (Tm) of mouse IgG1 CH2 is only 41°C. The Tm of human IgG1 CH2 is 54°C, which is also low but higher than that of mouse IgG1 CH2 (55). In serial comprehensive studies, it was found that (i) different IgG subclasses have different stability and aggregation propensities due to the sequence variation of their CH2 domains, and IgG1 is the best one among all the four subclasses in general (56–59); (ii) Fc aggregation induced by low pH is firstly triggered by CH2 unfolding associated with the protonation of specific acidic residues (54, 60). Therefore, it is one of the major determinants for the unfolding of Fc-based molecules (61). Engineering of CH2 to increase its stability and aggregation resistance is expected to improve the physicochemical properties of Fc, which could be used to modify Fc-based therapeutics.

CH3 is the Ig domain following CH2. The structure of CH3 is very similar to that of CH2 (Figure 1). It also contains seven β-strands from A to G connected by three loops (loops BC, DE, and FG) and two helices (helix 1 and 2) with a native disulfide bond between strand B (C367) and F (C425) as CH2 although strand D seems to be shorter than that in CH2 according to the crystal structure (Figure 1) (47, 51). Distinguishing from two CH2 domain with in Fc, two CH3 domains can interact with each other very strongly, which leads to the formation of dimeric Fc structure. Probably due to the homo-interaction, dimeric CH3 has much higher stability than monomeric CH2 (62). In the interface of two IgG1 CH3 domains, there are at least six residues (T366, L368, P395, F405, Y407, and K409) involved in the interactions (Figure 1) (63). In detail, residues T366 and Y407 form a hydrogen bond and represent the principal intermolecular and intramolecular contact with each other, while residue K409 forms a hydrogen bond with residue D399 on the partner CH3 domain. Residues L368 and F405 form intermolecular and intramolecular contacts through van der Waals interactions only. These five residues locate on the two internal antiparallel β-strands and form a patch at the center of the interface. P395 contributes to the flexibility of the proline-containing loop constituting the domain–domain interface. Furthermore, the direct evidence showed that the native disulfide bond is not only involved in the folding of single CH3 domain, but also related to the dimerization process of two CH3 domains (64, 65), which is helpful for prevention of aggregation (66). Combination of display technology and high-throughput sequencing discloses a stability landscape of the CH3 domain (67). For example, it has been found that hotspots locate at C- and F-strand (positions 378–383 and 423–428, respectively) which are tolerant and intolerant to substitution due to their different orientations in a β-sheet. The side chains of hydrophobic residues V379, W381, F423, and V427, as well as the disulfide bond forming residue C425, are directed to the hydrophobic core of the CH3 domain and interact with residues of the inner β-sheet. They are highly intolerant to mutation. By contrast, the solvent-exposed side chains of A378, E380, E382, S424, S426, and M428 are more tolerant to mutation. This strongly suggests that the intolerance to mutation of a particular residue is not primarily caused by its localization in a secondary structural element, but by side-chain interactions with other parts of the molecule. The factors are more complicated for those residues in the inner β-sheet because they interact with other residues either in other chains in the same domain or in the same chains in the symmetrical partner CH3 domain.

Interestingly, although the glycan chain does not contact the CH3 domain, the CH3 domain in glycosylated Fc reveals a higher stability than that in aglycosylated Fc, implying an indirect contribution of the glycan chain to maintain the CH3 structure domain possible through stabilization of CH2 domain (68). Although the CH2 domain firstly unfolds during acid-induced unfolding, the colloidal stability of the CH3 homodimer in the fully unfolded state is lower than that of CH2 monomer and aglycosylated Fc, and the unfolded CH3 homodimer forms much larger aggregates (68, 69). Therefore, it was concluded that (1) the unfolding process of CH2 and CH3 domains is independently from each other in the aglycosylated Fc region; (2) the colloidal stabilities of the CH2 and CH3 domains affect the aggregation process of the unfolded aglycosylated Fc region in a compensatory manner; and (3) the CH3 domain plays the most critical role among different Ig domains in driving intact antibody aggregation under acidic conditions (68, 69).

Although different antibody subclasses and subtypes have different Fc sequences, Fc is the common part of all the full-size antibodies and Fc-fusion proteins. To increase the stability and aggregation resistance of Fc, most previous work has focused on introducing mutations to form covalent interactions such as disulfide bonds or enhance the non-covalent interactions. In addition, change of the glycosylation form may also alter the folding of the Fc-based molecules.

### Introduction of Covalent Bond in Fc

Disulfide bonds are main kind of covalent interactions in intra- or inter-Ig domains, formed by the oxidation of two thiol groups within cysteine residues, which fix and stabilize the structure of proteins in an oxidative environment. There are 12 intra-domain disulfide bonds (each domain has one intra-domain disulfide bond locating in hydrophobic core) that exist in IgG, while 4, 6, 13, and 4 inter-chain disulfide bonds exist in IgG1, IgG2, IgG3, and IgG4, respectively, due to different length of hinge regions (70). Disruption of the intra-domain disulfide bonds in CL, VH, VL or CH3 results in a significant reduction of the structural stability of those domains, especially the thermodynamic stability (66, 71–77). Similarly, inter-domain disulfide bonds also have an effect on structural stability of IgG (68, 78). The existence of one disulfide bond in hydrophobic core of IgG domain could stabilize the structure. Hence, additional disulfide bond may improve the stability of Ig domain. The IgG1 Fc fragment consisting of two CH2 domains and two CH3 domains has four inherent intra-domain disulfide bonds (one disulfide bond in each domain). Additional disulfide bonds could be engineered in single domain or between two domains.

#### Engineering of Disulfide Bond in CH2

**Figure 2:**
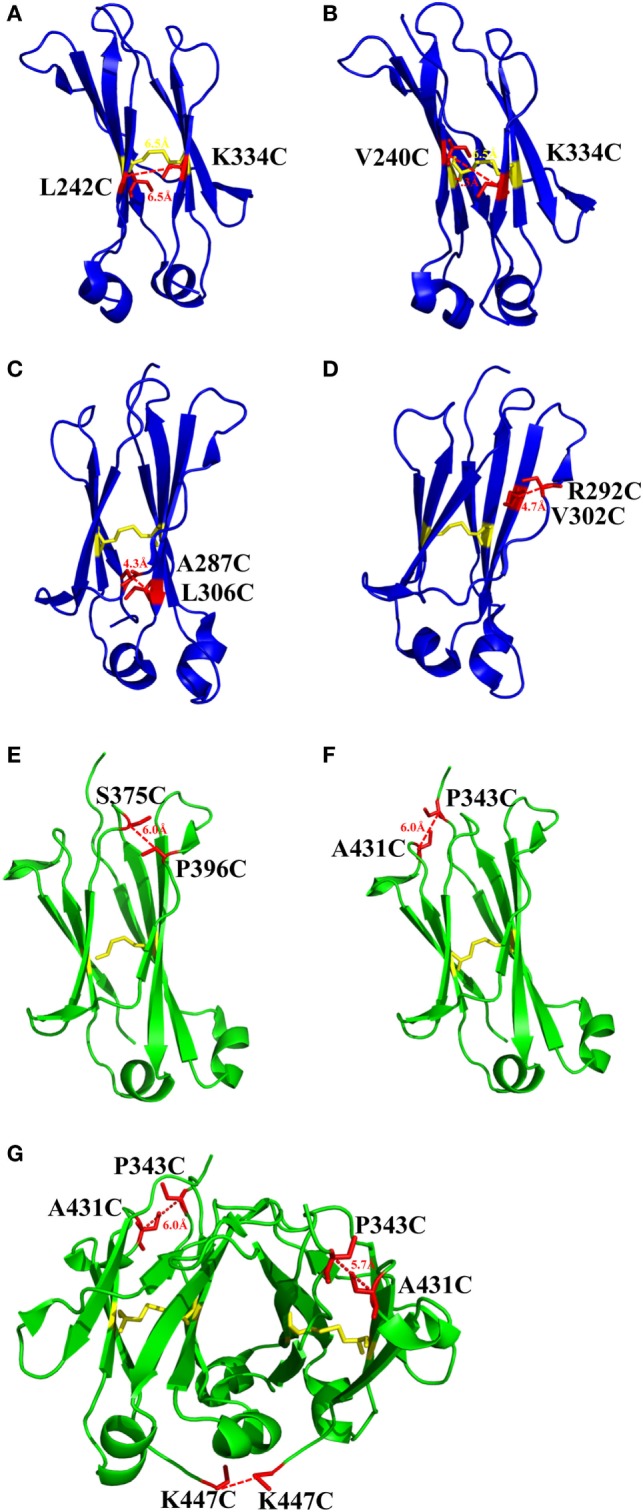
The structures of CH2 and CH3 domains [PDB 3AVE (47)] with different mutations for introduction of additional disulfide bonds presented by PyMOL. **(A)** CH2 mutant L242C/K334C. **(B)** CH2 mutant V240C/K334C. **(C)** CH2 mutant A287C/L306C. **(D)** CH2 mutant R292C/V302C. **(E)** CH3 mutant S375C/P396C. **(F)** CH3 mutant P343C/A431C. **(G)** CH3 mutant P343C/A431C/K447C. The native disulfide bond is colored by yellow, whereas the mutated residues for additional disulfide bonds are colored by red. All the marked distance is the measured between two α-carbon atoms in related two cysteines after mutagenesis by using PyMOL.

There is one intrinsic disulfide bond in hydrophobic core of CH2 domain between β-strand B and F (C261 in strand B and C321 in strand F) as mentioned earlier (Figure 1). In our previous research, based on possible distance of forming disulfide bond between two cysteines, five pairs of amino acids were substituted to cysteines among which two of them could be well expressed in bacterial expression system (55). Those two mutants termed m01 and m02, respectively, in which an additional disulfide bond between β-strand A and G was engineered by mutations on L242C and K334C in m01 (Figure 2A) as well as V240C and L334C in m02 (Figure 2B). The Tm of m01 is 73.8°C, which is almost 20°C higher than that of wide-type CH2 (wtCH2). Both of them are also much more stable against urea unfolding compared with wtCH2. Meanwhile, the secondary structure of m01 is not affected by this additional disulfide bond measured by circular dichroism (CD) and nuclear magnetic resonance. The stability of m02 is also much better than that of wtCH2, but the aggregation-resistant property is not as good as m01. It has been shown that the Tm of CH2 with mutations in L242C and L334C in IgG1 is 8.7°C higher than that in wtIgG1 (79). In another research (80), to improve the stability of an IgG1 variant with mutation of N297G (mAbW.IgG1, an effector function silenced IgG1), an additional disulfide bond according to the design of m01 was introduced between position L242 and K334 (81). As expected, the stability of this IgG1 variant (mAbW.SEFL2.0) with mutations on L242C/N297G/K334C is improved particular in the thermal stability, but it shows faster clearance in the rat in pharmacokinetics study. Another four IgG1 variants with different additional disulfide bond formation were designed, constructed, and expressed in CHO expression system, among which two variants mAbW.SEFL2.1 with mutations of A287C/N297G/L306C in CH2 domain (Figure 2C) and mAbW.SEFL2.2 with mutations of R292C/N297G/V302C in CH2 domain (Figure 2D) with improved stability, decreased rate of clearance, and longer half-lives in both the rat and cynomolgus monkey models compared with mAbW.IgG1. Importantly, the Tm values of mAbW.SEFL2.1 and mAbW.SEFL2.2 are about 8°C higher than that of mAbW.IgG1, while mAbW.SEFL2.0 is about 2°C higher than that of mAbW.IgG1, which indicates introduction of disulfide bonds in different positions results in different outcomes.

#### Addition of Disulfide Bond in CH3

The same as CH2 domain, CH3 domain also has one intrinsic disulfide bond in hydrophobic core (C367 and C425). To investigate the influence of this disulfide bond in CH3, a variant of CH3 reducing disulfide bond was engineered (66). And in the following studies it has been shown that the thermal stability of the reduced form is lower than that of the oxidized form measured by CD and differential scanning calorimetry (DSC) results. In addition, the reversibility after unfolding is also significantly lower. And although most of reduced form retained a stable dimeric structure, aggregation is improved. Similarly to CH2, introduction of additional disulfide bond inside the domain could also improve the stability of CH3. In another study, an additional disulfide bond was engineered into isolated monomeric CH3 by mutation of P343 and A431 to two cysteines, resulting in improved protein expression (up to fivefold) and elevated Tm (from 40.6 to 76.0°C), without affecting FcRn binding ability (82). In a more previous study, two additional disulfide bonds were engineered into CH3 domain, respectively. Hence the Tm value of Fc variant with dual mutations of P343C/A431C and S375C/P396 in CH3 domain (Figures 2E,F, respectively) was obviously improved measured by CD and DSC experiment compared with wtFc (83). Since two CH3 domains interact non-covalently with each other, in addition to introduction of intra-domain disulfide bonds, there are some works on engineering of additional inter-domain disulfide bonds between two CH3 domains. The last three amino acids (P445, G446, and K447) at C-terminal of CH3 were replaced by G, E, and C, respectively, derived from CL domain (Figure 2G), which could introduce an additional inter-domain disulfide bond between two CH3 domains in Fc or dimeric CH3 (68, 84). And the conformational stability of both the CH2 and CH3 domains could be improved in an Fc variant with this kind of inter-domain disulfide bond (68). In another research, an Fc variant (P343C, A431C P445G, G446E, and K447C) with not only additional intra-domain but also inter-domain disulfide bond could increase the thermostability of CH2 and CH3 (84). The Tm of CH3 in this variant was improved by 18.1°C compared with the wtFc. Furthermore, the properties of pH and aggregation resistance of this variant were also increased when compared with the wtFc. The mutations in CH2 and CH3 domains for introducing disulfide bonds are listed in Table 1.

**Table 1:** Introduction of disulfide bonds and engineering of non-covalent interactions in CH2 and CH3 domains.

Position	Domain^a^	ΔTm (°C)	Description^b^	Reference
**Covalent**				
L242C/K334C	CH2	18.7	Isolated IgG1 CH2	(55)
V240C/L334C	CH2	11.2	Isolated IgG1 CH2	(55)
L242C/K334C	CH2	8.7	CH2 in IgG1	(79)
L242C/K334C	CH2	2.1	CH2 in aglycosylated IgG1	(81)
A287C/L306C	CH2	7.8	CH2 in aglycosylated IgG1	(81)
R292C/V302C	CH2	8.1	CH2 in aglycosylated IgG1	(81)
P343C/A431C	CH3	35.4	Isolated IgG1 CH3	(82)
P343C/A431C	CH3	10.2	CH3 in IgG1 Fc	(83)
S375C/P396C	CH3	4.7	CH3 in IgG1 Fc	(83)
P343C/A431C	CH3	15.2	CH3 in IgG1 Fc	(83)
S375C/P396C				
P445G/G446E/K447C	CH3	3.5	CH2 in IgG1Fc	(84)
		9.1	CH3 in IgG1 Fc	(84)
P343C/A431C	CH3	14.5	CH2 in IgG1 Fc	(84)
P445G/G446E/K447C		18.1	CH3 in IgG1 Fc	(84)

**Non-covalent**				
L235K/L309K	CH2	2.7	CH2 in IgG	(45)
L234F/L235Q K322Q/M252Y S254T/T256E	CH2	5.7	Compare to IgG1 with Mutation of “TM-YTE”	(99)
G197K/S207G/T246L	Bovine CH3	10.0	Compare to bovine wtCH3	(102)
Q295F/Y296A	CH2	3.2	CH2 in IgG1 Fc	(103)
Truncation of N-terminal residues “APELLGG”	CH2	5.1	Isolated IgG1 CH2	(107)

*^a^All the domains are from human IgG1 if not specified*.

*^b^All the islolated domains are expressed in Escherichia coli, others are expressed in eukaryotic cells*.

### Optimization on Non-Covalent Interaction in Fc

Although introduction of disulfide bonds can significantly increase the stability of a protein, the risk of increasing aggregation propensity caused by incorrectly paired cysteines might not be neglected. Therefore, optimization of non-covalent interactions is another efficient strategy to improve the stability or aggregation resistance of Fc fragments. The optimization could be performed by site-directed mutations (without forming covalent disulfide bond) under the aid of sequence and structural information, and computation, which could change the local residue-to-residue interactions and influence the whole Fc-based molecule.

#### Introduction of Intramolecular Non-Covalent Interactions in CH2

**Figure 3:**
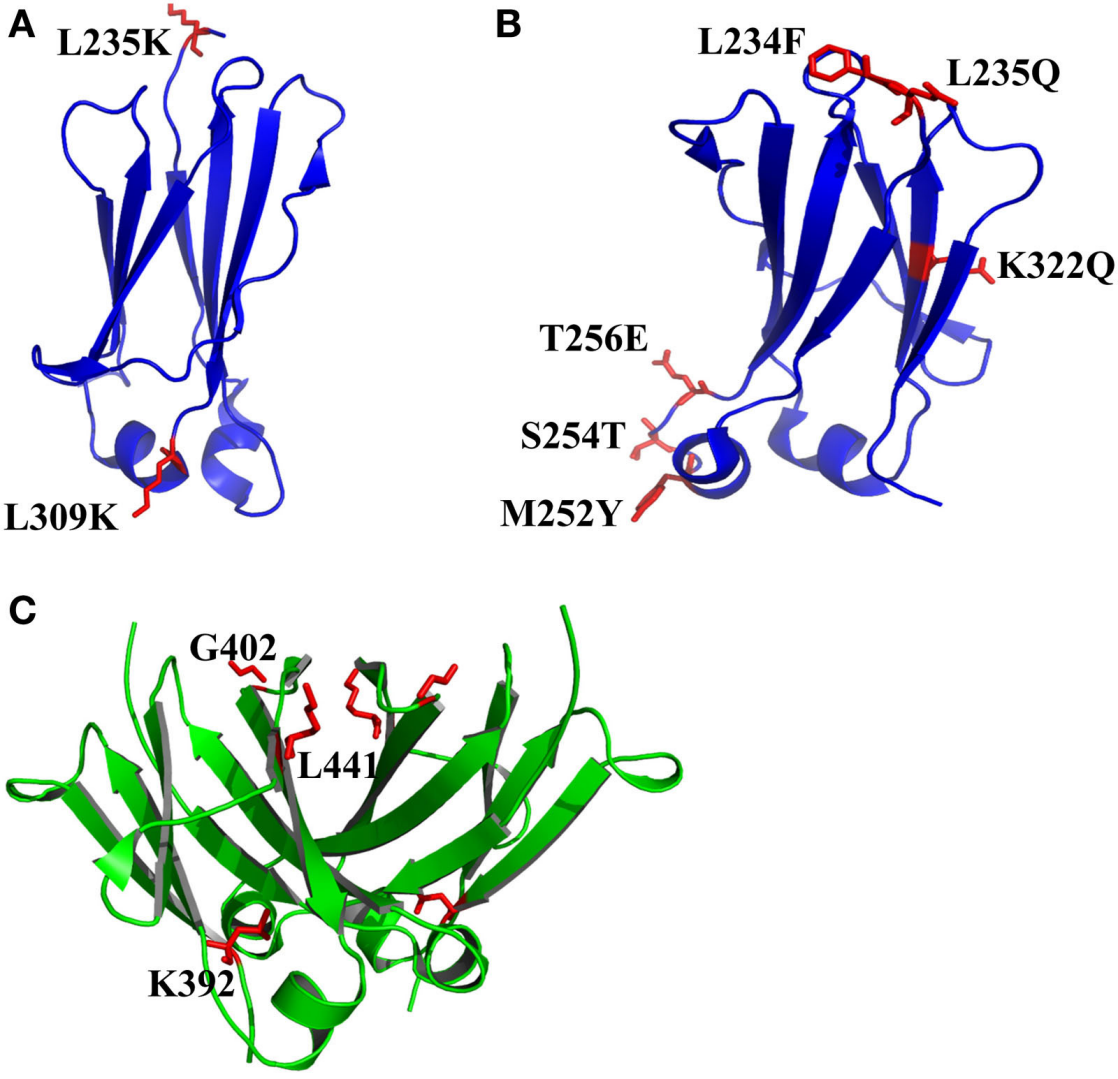
The structures of CH2 and CH3 domains [PDB 3AVE (47)] with optimized non-covalent interactions presented by PyMOL. **(A)** CH2 L235K/L309K mutant. **(B)** CH2 L234F/L235Q/K322Q/M252Y/S254T/T256E mutant (FQQ–YTE). **(C)** The residues K392, G402, and L441 in human IgG1 CH3 domain, which can be used to replace the corresponding residues in the bovine CH3 domain (G197, S207, and T246) for increase of the stability. The mutated residues for additional disulfide bonds are colored by red.

The exposure of hydrophobic residues may increase the aggregation propensity due to non-specific hydrophobic interactions, especially when the large hydrophobic clusters form. Therefore, rational disruption of those continuous hydrophobic residues without affecting the molecular structure may be helpful for decreasing the formation of aggregation. A computational technology termed spatial aggregation propensity (SAP) was developed to measure the dynamic exposure of hydrophobic patches and identify the location and size of these aggregation-prone regions based on the atomistic molecular dynamics simulations, which can guide the performance of target mutations for engineering of protein stability and aggregation resistance (45, 85, 86). By this technology, several mutations with different combinations were introduced to generate several IgG variants (45). One variant with combinational mutations of L235K and L309K in the CH2 domain (Figure 3A) showed not only increased thermostability stability but also improved aggregation resistance. Being a hydrophilic amino acid, lysine can discontinue the hydrophobic patch and reduce the non-specific interactions between hydrophobic patches. Furthermore, as mentioned earlier, CH2 domain is typically the least stable domain in the Fc portion, which influences the whole molecular stability and aggregation propensity. Therefore, the physicochemical properties of the antibody molecule could be optimized after rational introduction of proper hydrophilic amino acids for disruption of the large hydrophobic aggregation-prone clusters. In addition to SAP (87–89), several online programs such as TANGO^3^ (90–92), PASTA^4^ (93), AGGRESCAN^5^ (94), and Aggrescan3D^6^ (95) are also widely used to predict aggregation-prone regions within proteins. Therefore, it is desired that new Ig variants could be identified in the future.

In many instances, it might be desirable for a therapeutic mAb and Fc-fusion protein to lack/reduce effector functions. According to previous studies, two sets of mutations in CH2 domain including “TM” (triple mutations of L234F/L235E/P331S) and “YTE” (M252Y/S254T/T256E) are effective in abolishing the binding of Fc to FcγRs (96) and C1q and enhancing the pH-dependent binding to FcRn (97, 98), respectively, which can generate an IgG variant (TM–YTE) with extended half-life but without Fc-mediated effector functions *in vivo*. However, these mutations adversely influence the stability and aggregation propensity and result in many difficulties for further applications (99). For example, it has been shown that replacement of the original amino acids M252, S254, and T256 to Y, T, and E significantly reduced the stability and increased the aggregation tendency due to the increased local flexibility of the 244–254 segment in CH2 (100, 101). To compensate the decreased physicochemical properties, several mutants were designed with different combinations of mutations with the strategies including substitution of charged residues (e.g., E) by uncharged residues (e.g., Q). According to a serial of experiments by characterization of designed mutants, one novel mutant (FQQ–YTE) with combinational mutations of L234F/L235Q/K322Q/M252Y/S254T/T256E (Figure 3B) was identified, which had significantly improved conformational stability while retaining the same biological activities as TM–YTE mutant (99).

#### Introduction of Intra- and Intermolecular Non-Covalent Interactions in CH3

Sequence analysis among different Ig classes and subtypes can provide useful clues for Fc optimization. One good example is the mutation on bovine IgG1 CH3 for increased stability deduced from the sequence and structural information (102). According to sequence alignment and frequency analysis among 36 unique IgG Fc sequences originating from 19 different mammalian species, bovine IgG1 demonstrated the closest resemblance to the consensus sequence other than primate IgG sequences, while a few crucial positions could be mutated to make the molecule more stable. Therefore, the residues S174, Y179, G197, S207, and T246 in bovine IgG1 CH3 numbered according to the reference (102) which are partnered with G371, D376, K392, G402, and L441 in human IgG1 CH3 were selected to be substituted by G, D, K/A, G, and L, respectively. Four of these 5 mutant positions are at residue sites of reasonable heterogeneity (i.e., the measured positional entropies are in the top 70% for all residue positions), while mutation at S207 (G402) was the only position that highly conserved because only 4 of the 36 sequences have another residue other than G. In spatial structure, each residue is far away from the four others. After measurement of heat-induced unfolding, one combination with G197K/S207G/T246L (Figure 3C) showed the highest Tm, which increased about 10°C compared with that of wide-type bovine IgG1 CH3. The experimental results could be explained as follows. First, among these mutations, replacement of G197 to K392 inserts a lysine at the interface between the CH3 dimer locating at the hydrophobic portion of the side-chain that packs against a hydrophobic patch created by F210 (F405) and V202 (V397) of the opposite dimer subunit. Although adding a hydrophobic moiety like alanine to this cavity is also better than original G, introduction of positive charged lysine at this position is shown to be best due to the adjacency to D204 (D399), a negative charged amino acid can possibly enhance the favorability of lysine at this position. Second, substitution of S207 by G402 places G in a tight turn between β-strands, which likely releases strain induced by the tight turn since the backbone dihedral angles of this residue in the crystal structure are outside the generally allowed ranges for α-substituted amino acids (all amino acids other than G). Finally, the mutation of T246 to L441 completely buries the isobutyl side chain into the hydrophobic interior of the β-sandwich. Although burial of three groups are expected for more favorable contributions, the actual effect is not as obvious as desired. The reason is that the side chain of T can compensate somewhat for the loss of buried hydrophobic surface area due to steric placement or hydrogen bonding.

This finding indicates that cross-species analysis provides useful information about the relationship between residues and physicochemical properties, and further guides the rational design for a better Fc with more stable, more aggregation-resistant and more soluble characteristics.

#### Engineering of Loop and Other Regions in CH2 and CH3

**Figure 4:**
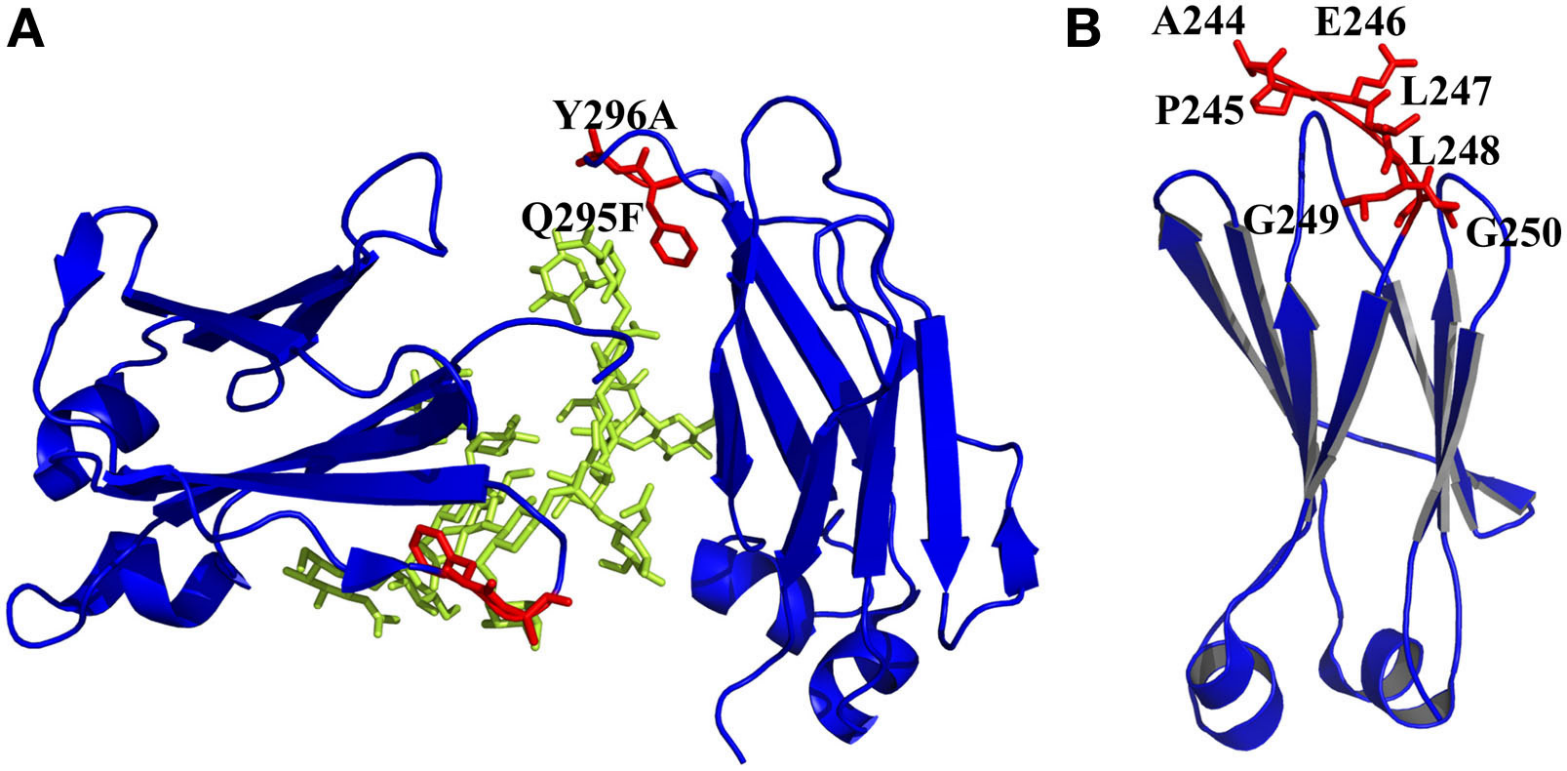
Engineered of loop and other regions in CH2 presented by PyMOL. **(A)** Introduction of an enhanced aromatic sequon (EAS) (Q295F/Y296A) in CH2 loop DE [PDB 3AVE (47)]. The mutated residues are colored by red, whereas the oligosaccharides are colored by lemon color. **(B)** Truncation of N-terminus of CH2. Seven unstructured residues from A244 to G250 are colored by red, which could be removed [PDB 1HZH (51)].

Both CH2 and CH3 domains have several flexible loop regions, which could also be the targets for optimization of the physicochemical properties. For example, to stabilize the CH2 domain, an enhanced aromatic sequon (EAS) (Q295F/Y296A) (Figure 4A) was engineered into the top of *N*-glycosylated DE loop, which led to a 4.8°C increase of the Tm of the purified IgG1 Fc fragment (103). This strategy could be used in optimizing a full-length IgG1 molecule for enhancement of its resistance to unfolding and aggregation. The crystal structure of the EAS-stabilized IgG1 Fc fragment reveals the importance of the GlcNAc1·F295 interaction, as well as the participation of the core fucose (Fuc) attached to GlcNAc1 in an interaction with F295. As mentioned earlier, the yeast display technology is also used in high-throughput screening of improved CH3 mutants with replacement of its original loops for optimization on Fc and development of antigen-specific Fc binders [Fc-antigen binding (Fcab)] (104, 105).

It has been shown that natural β-sheet proteins use negative design to avoid edge-to-edge aggregation, which indicates that the residues at N- and C-termini may be involved in aggregation formation (106). A shortened CH2 (CH2s) (Figure 4B) was constructed with truncation of seven unstructured N-terminal residues according to a crystal structure of an intact IgG1 (PDB 1HZH) (51), which showed significantly increased aggregation resistance and potential Fc-mediated functions (107). Engineering of C-terminal residues in CH2 might also have the same influence as described earlier. The optimizations on non-covalent interactions in CH2 and CH3 domains are summarized in Table 1.

### Glycoengineering in Fc

There are only two symmetrical N-glycosylation sites in the Fc fragment, which locate at amino acid position N297 in IgG1 CH2 domain. It has been widely accepted that N-glycan has a critical impact on the structure and effector functions of Fc-based therapeutics (108–111). Absence of the N-glycan can cause dramatic conformational change and decreased stability of the Fc (112–117). Without N-glycan, the binding of Fc to various receptors and their associated biological functions is either reduced or completely lost (112, 116, 118–120). The factors influencing the oligosaccharide profiles of Fc-based therapeutics are very many, such as the use of different cell lines, cell culture conditions, scales, and other factors (121, 122). Therefore, variation of glycoform is one of the main factors that cause heterogeneity of therapeutic antibodies and Fc-fusion proteins. Although aglycosylated Fc-based therapeutics have been explored for clinical use, the majority of these therapeutic proteins are still glycosylated. In most conditions, the function of N-glycan is irreplaceable for the treatment of some diseases.

**Figure 5:**
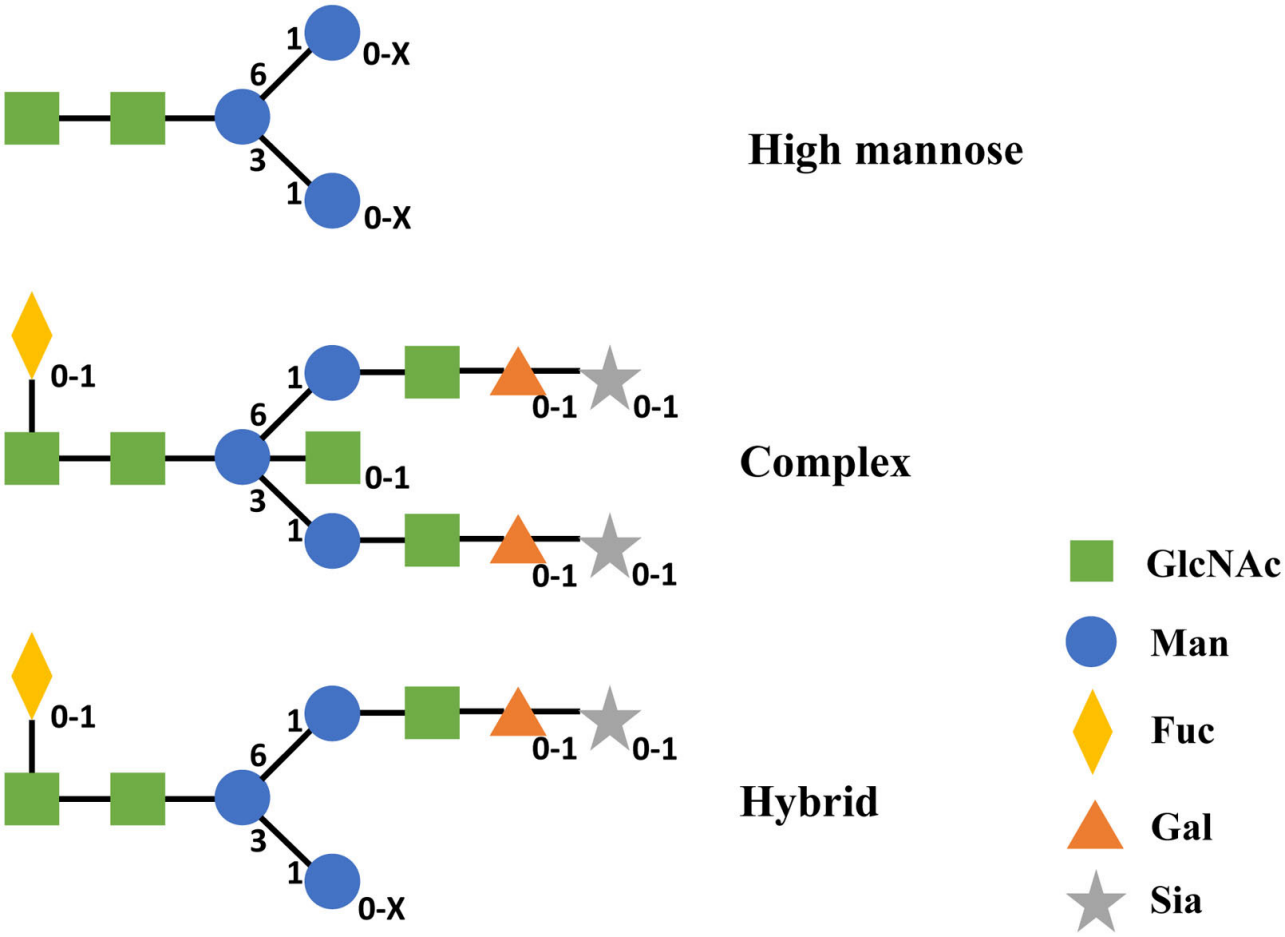
Structures of three categories of N-linked oligosaccharides in IgG1 CH2. Subscripts indicate the absence (0) or presence (1 and *X*) of corresponding monosaccharide. *X* represents a number that is equal or greater than 1 of this monosaccharide. Abbreviations: Gal, galactose; GlcNAc, *N*-acetylglucosamine; Man, mannose; Fuc, fucose; Sia, sialic acid.

According to the difference of oligosaccharides on the outer arms, N-glycan can be classified into three categories including high mannose (Man), complex, and hybrid (49). All of these classes share an invariable core structure containing two copies of primary *N*-acetylglucosamine (GlcNAc), one primary Man and the two secondary Man residues. High Man oligosaccharides are composed of Man only in the outer arms. Complex oligosaccharides are composed of GlcNAc and galactose and potentially sialic acid in the outer arms. Meanwhile, complex oligosaccharides can exist with or without the core Fuc. Hybrid oligosaccharides are made of one arm with complex and the other arm with high Man residues (Figure 5) (49). Therefore, different configurations and compositions of N-glycan can lead to >400 different variants concerning the two CH2 domains of IgG Fc (123).

The position of glycosylation on the Fc determines that it has no impact on antigen–antibody binding and FcRn binding, but oligosaccharides are critical for the binding of FcγRs and C1q, which trigger different immune responses (FcγRs for ADCC and C1q for CDC). On the other hand, changes occurring on the composition of N-glycan can influence the conformation of the whole antibody or Fc-fusion protein molecule, causing alteration in the binding affinity for various FcγRs (124). That is one of the reasons for the glycosylation playing such effective role in engineering Fc-based therapeutics.

The process of glycosylation only occurs in eukaryotes, so *Escherichia coli* and Chinese Hamster Ovary cell (CHO) derived IgG1 Fc were usually used to illustrate the impact of N-glycosylation on the stability of Fc, representing aglycosylated and glycosylated Fc proteins, respectively (50). Both of them behave similarly during heat and low pH induced unfolding. First, the tertiary structure and CH2 domain are unfolded, then the secondary structure and CH3 domain are changed. Due to the interaction of oligosaccharides, the glycosylated Fc protein is more compact (smaller hydrodynamic radius) than the aglycosylated Fc protein at neutral pH. In the aspects of thermostability and pH resistance, the Tm of glycosylated CH2 domain is 4–5°C higher than that of aglycosylated domain and the acid resistance of glycosylated Fc is ~0.5 pH lower than aglycosylated Fc (50).

Previous studies have proved that truncation of N-glycosylation is a major method to understand the relationship between the functions, structures and Fc glycoforms. Fc glycoform variants with partial or complete removal of glycan carbohydrates were compared with wtFc by using long molecular dynamics simulations (125). The results indicated that glycan truncation or removal can cause quaternary structural deformation of the Fc due to the disruption or loss of a lot of inter-glycan contacts. Because the existence of the weak binding through two oligosaccharides, glycan truncation/removal can also cause the tertiary structural deformation of CH2 domains, which results in destabilization of individual CH2 domains. During elevating Tms, glycan truncation is differentially affecting structural deformation in locations of helices 1 and 2 in CH2 that are far from the oligosaccharide attachment point. Deformation of these helices, which form part of the binding surface to FcRn, could affect the binding to FcRn if these regions are unable to refold after Tm normalization. During elevated Tm simulations of the deglycosylated variant, CH2 domains collapse onto CH3 domains. All these studies show that glycosylation plays an important role in the stability of Fc. Besides that, different formations of glycosylation in N297 site were compared, containing three Fc proteins produced from the yeast *Pichia pastoris* with three kinds of glycosylation sites (di-, mono-, and nonglycosylated) and another three different forms of nonglycosylated Fc (mutating N297 to two different amino acids and enzymatic digestion of the Fc glycoforms), to examine the differences of structural stability. Under different pH conditions, the di- and monoglycosylated forms of Fc showed the highest and lowest levels of stability, respectively, while the stability of nonglycosylated form was in the middle and depended on the solution pH (126, 127). Furthermore, hemi-glycosylated (same to monoglycosylation) Fc shows that the binding affinities toward all FcγRs were significantly decreased and a moderate decrease (~20%) in C1q binding, representing change of effector functions (128).

Lots of previous studies have proved that glycosylation also deeply affects the aggregation of antibodies and Fc-fusion proteins (126, 129–131). By comparing the differences between glycosylated and deglycosylated antibodies, it has been found that deglycosylated antibodies had not only less thermostability and resistance to GdnHCl-induced unfolding, but also higher aggregation rates at a accelerated stability study (129). The possible reason for these findings is that disruption of protein–carbohydrate interactions leads to the exposure of aggregation-prone motifs and further results in the aggregation of the whole deglycosylated antibodies (130). The interactions between two monomeric Fc regions of an antibody include protein–protein, carbohydrate–carbohydrate, and protein–carbohydrate, all these forces have contributions to the stability and aggregation resistance. So any change of oligosaccharides (e.g., truncation and variation on glycoforms) might have serious impact on the physicochemical properties of Fc-based therapeutics.

Although the importance of N-glycosylation on Fc is clear, the production of Fc-based proteins with selected glycoforms is still very difficult. For now, the enzymatic modification of recombinant IgGs *in vitro* or engineering the host expression system can be used to modify the glycoforms of Fc-based therapeutics (108).

Due to the complexity and limit of technology for engineering the N-glycoform, lots of work still focuses on the generation of IgG variants with aglycosylation, which only needs to introduce a mutation into N297, for decrease of the effector functions to treat some chronic diseases. However, as described earlier, these mutants may influence the physicochemical properties of Fc-based proteins deeply and cause the loss of efficacy, which will be further discussed in the following paragraph.

## Relationships Between Physicochemical Properties and Clinical Potential

The development of therapeutic antibodies, Fc-fusion proteins, and Fc-based antibody domains faces many challenges from bench to bedside. For example, an mAb may have excellent activities *in vitro* but lose the entire or major function *in vivo* and *vice versa*. Hence, to make a successful Fc-based therapeutics, it may need many modifications before clinical use.

Historically, either IgG2 or IgG4 isotypes have been selected for applications where cytotoxic effector functions are not required due to their limited cytotoxic effector functions (132). However, IgG1 is still the first choice due to its comprehensive advantages in many cases. Therefore, to eliminate undesired effector functions, removal of the glycosylation is an efficient way. A major concern is that deglycosylation will lead to instability and aggregation. Hence, the IgG1 scaffold with stable effector functions missing (81) needs to be further optimized. Based on the computational modeling, two variants mAbW.SEFL2.1 and mAbW.SEFL2.2 with tri-mutations on A287C/N297G/L306C and R292C/N297G/V302C, respectively, as mentioned earlier showed improved stability, decreased clearance rate, and longer half-life (80), which could be used for development of therapeutic mAbs without effector functions.

As mentioned, Etanercept is one of the most successful recombinant Fc-fusion proteins in the market. When administered *in vivo*, the dose of each injection is large (e.g., 25 mg/twice or 50 mg/once per week), which requires high concentration of Etanercept in the formulation. The risk of aggregate formation in this condition is increasing, which may lead to loss of functions and increase of side effects. To improve the solubility and reduce the aggregation, two mutations D239E and L241M were introduced into the Fc portion of Etanercept, which may increase the stability and aggregation resistance of whole recombinant protein (133). Actually, the formation of aggregation should be seriously considered when developing a biosimilar of Etanercept (134).

As a domain from Fc, CH2 has been proposed as a scaffold for development of C-based single domain antibody (C-sdAb), which has been extensively reviewed (10, 28). CH2 could have or partially have Fc effector functions and affinity to FcRn. Therefore, it could have relatively longer serum half-life after engineering due to the containment of FcRn binding sites inherited from Fc (135, 136) compared with other Ig domains (e.g., VH) as binders (29). Based on this scaffold, a panel of C-sdAbs has been selected against different targets (137, 138). However, in general, their activities are modest. Since it has been shown that CH2-based binders tend to aggregate, a mission for development of clinically potential C-sdAbs is to further modify the scaffold to increase its stability and decrease its aggregation propensity (55, 107, 135, 139, 140), as well as for development of other engineered domains such as Fcab (104, 105, 141), monomeric Fc (142) and monomeric CH3 (82) derived from Fc fragment. Although preliminary results show the proof-of-concept, there is still very long way to achieve the final aim.

## Summary and Prospective

The problems caused by instability and aggregation propensity are major restrictions in Fc-based biopharmaceutical industry. Many other reviews have already been focused on these issues (32, 46, 143). In this review, we summarized and discussed current status in the related field in Fc engineering. Although lots of work has been done to increase the stability and aggregation resistance of Fc, it seems that the *in vivo* outcomes are not very clear after optimization. On the other hand, plenty of work has also been done to enhance the Fc-mediated effector functions and extend the half-life. However, the value for clinical application is not well verified. At present the fact is that only a few candidates among many finally enter the market due to various reasons. Therefore, more attentions should be paid toward the relationship between physicochemical optimizations and potential clinical applications, which may accelerate the development of Fc-based therapeutics.

## Author Contributions

RG designed the review article topic and text structure; CY, XG, and RG wrote the manuscript.

## Conflict of Interest Statement

The authors declare that the research was conducted in the absence of any commercial or financial relationships that could be construed as a potential conflict of interest.

## Footnotes

^1^http://www.antibodysociety.org/tag/approved-antibodies/.

^2^http://investors.amgen.com/phoenix.zhtml?c=61656&p=irol-reportsannual.

^3^http://tango.crg.es/.

^4^http://protein.bio.unipd.it/pasta2/index.html.

^5^http://bioinf.uab.es/aap/.

^6^http://biocomp.chem.uw.edu.pl/A3D/.
